# The mitochondrial genomes of the ciliates *Euplotes minuta *and *Euplotes crassus*

**DOI:** 10.1186/1471-2164-10-514

**Published:** 2009-11-06

**Authors:** Rob M de Graaf, Theo A van Alen, Bas E Dutilh, Jan WP Kuiper, Hanneke JAA van Zoggel, Minh Bao Huynh, Hans-Dieter Görtz, Martijn A Huynen, Johannes HP Hackstein

**Affiliations:** 1 Department of Evolutionary Microbiology, IWWR, Radboud University Nijmegen, Heyendaalseweg 135, 6525AJ Nijmegen, The Netherlands; 2 Center for Molecular and Biomolecular Informatics, Nijmegen Center for Molecular Life Sciences, Radboud University Nijmegen Medical Centre, Geert Grooteplein 28, 6525GA Nijmegen, The Netherlands; 3 Department of Zoology, Biological Institute, University of Stuttgart, Pfaffenwaldring 57, D-70569 Stuttgart, Germany; 4 CIHR Group in Matrix Dynamics, University of Toronto, 150 College Street, Toronto, Ontario, Canada M5S 3E2; 5 Laboratoire de Recherche sur la Croissance Cellulaire, la Réparation et la Régénération Tissulaires (CRRET), EAC 7149 CNRS, Université Paris EST, 61, Avenue du Général de Gaulle, 94010 Créteil Cedex, France

## Abstract

**Background:**

There are thousands of very diverse ciliate species from which only a handful mitochondrial genomes have been studied so far. These genomes are rather similar because the ciliates analysed (*Tetrahymena spp*. and *Paramecium aurelia*) are closely related. Here we study the mitochondrial genomes of the hypotrichous ciliates *Euplotes minuta *and *Euplotes crassus*. These ciliates are only distantly related to *Tetrahymena spp*. and *Paramecium aurelia*, but more closely related to *Nyctotherus ovalis*, which possesses a hydrogenosomal (mitochondrial) genome.

**Results:**

The linear mitochondrial genomes of the hypotrichous ciliates *Euplotes minuta *and *Euplotes crassus *were sequenced and compared with the mitochondrial genomes of several *Tetrahymena *species, *Paramecium aurelia *and the partially sequenced mitochondrial genome of the anaerobic ciliate *Nyctotherus ovalis*. This study reports new features such as long 5'gene extensions of several mitochondrial genes, extremely long *cox1 *and *cox2 *open reading frames and a large repeat in the middle of the linear mitochondrial genome. The repeat separates the open reading frames into two blocks, each having a single direction of transcription, from the repeat towards the ends of the chromosome. Although the *Euplotes *mitochondrial gene content is almost identical to that of *Paramecium *and *Tetrahymena*, the order of the genes is completely different. In contrast, the 33273 bp (excluding the repeat region) piece of the mitochondrial genome that has been sequenced in both *Euplotes *species exhibits no difference in gene order. Unexpectedly, many of the mitochondrial genes of *E. minuta *encoding ribosomal proteins possess N-terminal extensions that are similar to mitochondrial targeting signals.

**Conclusion:**

The mitochondrial genomes of the hypotrichous ciliates *Euplotes minuta *and *Euplotes crassus *are rather different from the previously studied genomes. Many genes are extended in size compared to mitochondrial genes from other sources.

## Background

Ciliates, unicellular eukaryotes, are extremely species-rich and colonize a very broad spectrum of ecological niches. They are characterized by complexes of cilia, used for swimming and food capturing and by a nuclear dimorphism that is unique for ciliates. All members possess a micronuclear genome, which is active in sexual reproduction, and a macronuclear genome that is transcriptionally active during somatic development and maintenance. In addition to the macronuclear and micronuclear genomes, aerobic ciliates also possess a mitochondrial genome. Although there are thousands of different ciliate species, only six mitochondrial genomes have been completely sequenced and analyzed thus far: *P. aurelia *[1] and five *Tetrahymena *species: *T. pyriformis, T. thermophila, T. pigmentosa, T. paravorax and T. malaccensis *[2-4]. Only minor differences between the mitochondrial genomes of the *Tetrahymena *species were found. The mitochondrial genomes of *P. aurelia *and *Tetrahymena *species are also very similar; only two large blocks of genes are shifted between them but within these blocks the gene order is conserved. A third smaller block, containing the split mitochondrial ribosomal *rnl *gene, is duplicated in *Tetrahymena *but not in *Paramecium*. Although most of the sequenced mitochondrial genomes are circular mapping, the mitochondrial genomes of *Paramecium *and *Tetrahymena *are monomeric linear and capped with telomeres. No mitochondrial genomes have been sequenced in the order of hypotrichous ciliates that contain intensively studied species such as *Oxytricha *and *Stylonichia *as well as *Euplotes*, a genus widely distributed in freshwater and seawater environments. The two *Euplotes *species studied here (*E. crassus *and *E. minuta*) are both marine ciliates that were collected in the Mediterranean sea.

**Figure 1:**
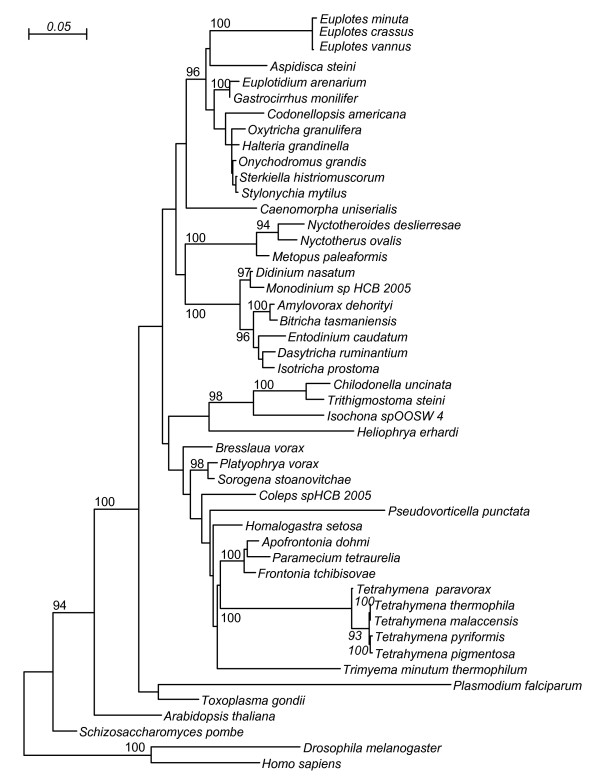
**Taxonomy of ciliates**. A maximum likelihood phylogeny from selected 18S rRNA genes. Only bootstrap values equal or larger than 90/100 are indicated.

We investigated the mitochondrial genome organization of *Euplotes *for three reasons: firstly, because *Euplotes *is only distantly related to *P. aurelia *and the various *Tetrahymena *species, the only species from which mitochondrial genomes have been studied so far (Figure 1). Secondly, because *Euplotes *contains two morphologically different types of mitochondria, which might possess different genomes [5,6] and thirdly, because we assumed that *Euplotes *is more closely related to *Nyctotherus ovalis *than *Tetrahymena *or *Paramecium*. Phylogenetic analysis, however, did not support this assumption because of lacking statistical support (Figure 1). Nevertheless, it is likely that its organellar genome is closely related to the hydrogenosomal genome of *Nyctotherus ovalis*, which exhibits characteristics of a ciliate mitochondrial genome and significant sequence similarity to certain *Euplotes *genes. *Nyctotherus ovalis*, which thrives in the hindgut of various cockroach species, has been investigated extensively, but only 14 kb of its hydrogenosomal genome have been sequenced so far [7-10]. Here, we show that the mitochondrial genomes of *E. crassus *and *E. minuta *are linear with a large repeat region in the middle that is potentially involved in transcription initiation. The gene content of the *Euplotes *genome is almost identical to that of *Paramecium *and *Tetrahymena*, but the order of the genes is completely different. We discuss the observation that *Euplotes *contains extremely large *cox *genes and several other mitochondrial genes with large extensions. It is shown that several N-terminal extensions of the mitochondrial genes have the potential to function as mitochondrial import signals.

## Results and Discussion

### Structure of the mitochondrial genomes

**Figure 2:**
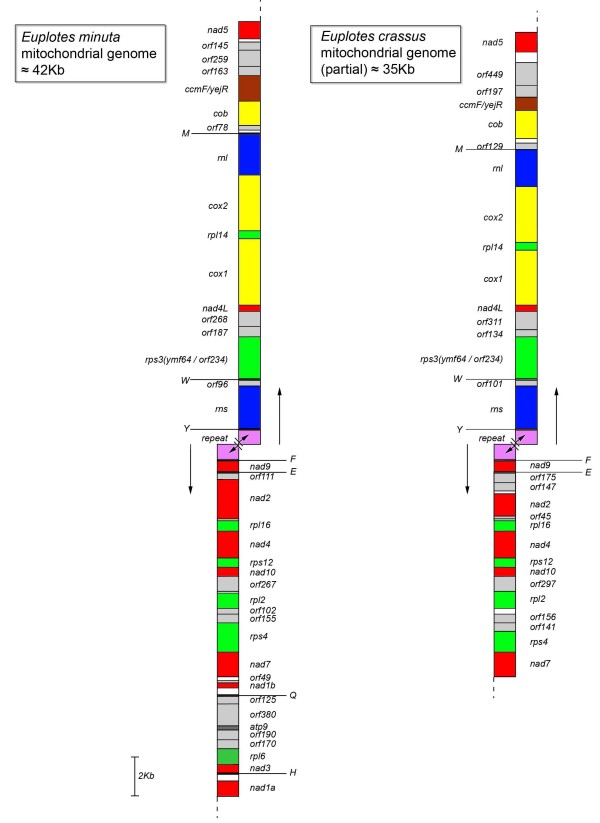
**Mitochondrial gene map of *Euplotes minuta *and *Euplotes crassus***. Red: Complex I genes, blue: rRNA genes, green: ribosomal proteins, yellow: Complex III and IV genes, grey: unidentified open reading frames, pink: repeat region, dark grey: *atp9 *gene, white: intergenic spacers. Capital letters indicate the various tRNA genes. Arrows: direction of transcription.

**Figure 3:**
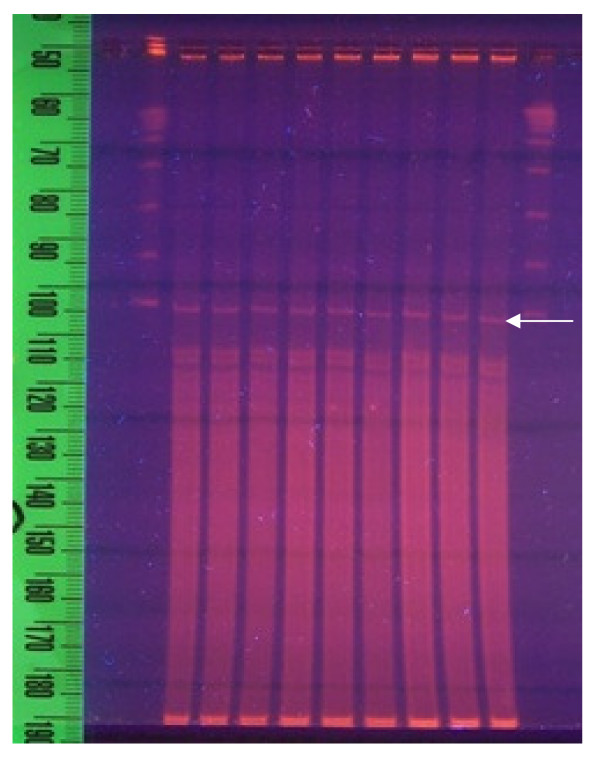
**Pulsed field gel electrophoresis of genomic DNA of *Euplotes minuta***. Lanes 1 and 11 contain lambda concatamer (marker). Lanes 2-10 contain genomic DNA of *Euplotes minuta*. The mitochondrial band (arrow) is located just below the one lambda band (48 Kb).

The linear mitochondrial DNA of *E. minuta *has been completely sequenced with exclusion of the telomeres and a repeat region of more then 1000 base pairs that is located almost in the middle of the mitochondrial genome. The length of the sequenced mitochondrial genome of *E. minuta *clearly exceeds 41600 bp, while 33273 bp (excluding the repeat region) of the mitochondrial genome of *E. crassus *have been sequenced (Figure 2). The length of the telomeres can only be estimated roughly since it is known from investigation of 5 different *Tetrahymena *species that the composition and length of mitochondrial telomeres can differ enormously [11,12]. Also, in three *Tetrahymena *species the terminal repeats at both ends of the mitochondrial DNA are completely different. Moreover, analysis of the mitochondrial genome of *T. malaccensis *has shown that telomeric lengths can vary between 700 and 4200 bp with an average size of 2600 bp. [12]. Since pulsed field gel electrophoresis of *E. minuta *DNA has indicated that the total length of the mitochondrial genome is clearly less than 48 Kb (Figure 3), it is likely that we have sequenced the total mitochondrial genome with exception of the telomeres. This interpretation is supported by the observation that chromosome walking using organelle DNA failed to provide evidence for the presence of additional DNA at the ends of the sequenced mitochondrial genome.

**Figure 4:**
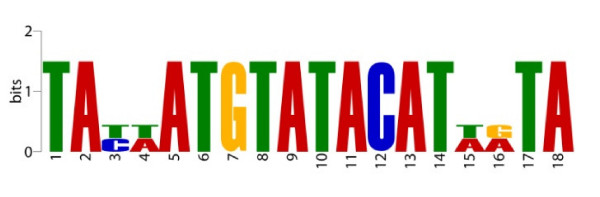
**Structure of the central repeat unit**. The repeat unit is palindromic except for the positions 3-4/15-16. It is identical in *E. crassus *and *E. minuta*.

The central repeat region is made up from 18-bp units that are palindromic except for the positions 3-4/15-16. The repeat units are identical in *E. crassus *and *E. minuta *(Figure 4).

Because the direction of transcription of all mitochondrion encoded genes is away from this repeat region (Figure 2), we tested whether the palindrome exhibits significant sequence similarity to any known transcription factor binding site using the online motif comparison tool Tomtom [13]. As expected, no significant levels of sequence similarity were found (E-values > 0.1). Notably, it has been observed that unrelated A-T rich repeat units serve as autonomously replicating sequences in the mitochondrial DNA of *Paramecium *and *Tetrahymena*; these units are located at one end of the mitochondrial chromosome, close to the telomeric repeats [14-16]. Other examples of repeat regions in mitochondrial genomes of protists are found in the cryptophyte algae *Rhodomonas salina *[17] and *Hemiselmis andersenii *[18]. Both mitochondrial genomes contain a large complex repeat region that seems to play a role in transcription. However, the mitochondrial genomes of these unicellular cryptophyte algae are not linear but circular mapping.

The overall A+T content of the mitochondrial genome of *E. minuta*, (64.0%), is much lower than in *T. pyriformis *(78.7%) but significantly higher than in *P. aurelia *(59.0%) [1]. Genes are tightly packed and the intergenic regions (4.1% of the genome) are generally short (ranging from 1 to 385 nucleotides, with an average size of 66 nucleotides). These intergenic regions have an overall A+T content of 68.9% which is hardly higher than in the coding areas. We found eight cases where the *orf*s overlap (9-96 bp.) and no gene duplication. One gene *(nad 1*) was split into two parts that are located on different positions of the genome.

The overall A+T content of the sequenced part of the mitochondrial genome of *E. crassus *is 65.3%. The genes in the mitochondrial genome of *E. crassus *are also tightly packed and intergenic spacers (4.2%) have a length of 2 to 413 nucleotides with an average size of 77 nucleotides and an A+T content of 68.4%. Overlapping *orf*s were identified in 12 cases with overlaps varying in size from 3 to 100 base pairs.

### The mitochondrial genes of *Euplotes minuta *and *Euplotes crassus*

The mitochondrial DNA of *E. minuta *includes 12 protein coding genes involved in the electron transport chain, 7 ribosomal protein coding genes, 2 ribosomal RNA genes, 7 transfer RNA genes, and one gene that encodes a cytochrome c assembly protein (*ccmF*/*jeyR*) (Table 1). Finally, 15 *orf*s were found with no detectable sequence similarity to known genes (Table 2). The sequenced part of the mitochondrial genome of *E. crassus *contains 10 genes of the electron transport chain, 6 ribosomal protein coding genes, 2 ribosomal RNA genes, 5 transfer RNA genes, the *ccmF*/*jeyR *gene and 11 *orf*s with significant sequence similarity to *E. minuta *genes, but no detectable sequence similarity to other known genes (Table 1, 2).

**Table 1:** Mitochondrion-encoded genes of *Euplotes minuta, Euplotes crassus *and other ciliates

*gene*	*E. minuta*	*E. crassus*	*T. pyriformis*	*P. aurelia*
***nad1***	*	ns	*	*
***nad2***	*	*	*	*
***nad3***	*	ns	*	*
***nad4***	*	*	*	*
***nad4L***	*	*	*	*
***nad5***	*	*	*	*
***nad6***	-	-	*	*
***nad7***	*	*	*	*
***nad9***	*	*	*	*
***nad10***	*	*	*	*
***rnl/lsu***	*	*	*	*
***rns/ssu***	*	*	*	*
***cob***	*	*	*	*
***cox1***	*	*	*	*
***cox2***	*	*	*	*
***atp9***	*	ns	*	*
***ccmF/yejR***	*	*	*	*
***rps3***	*	*	*	*
***rps4***	*	*	-	-
***rps12***	*	*	*	*
***rps13***	-	-	*	*
***rps14***	-	-	*	*
***rps19***	-	-	*	-
***rpl2***	*	*	*	*
***rpl6***	*	ns	*	-
***rpl14***	*	*	*	*
***rpl16***	*	*	*	*
***trnE(Glu)***	*	*	*	-
***trnF(Phe)***	*	*	*	*
***trnH(His)***	*	ns	*	-
***trnL(Leu)***	-	-	*	-
***trnM(Met)***	*	*	*	*
***trnW(Trp)***	*	*	*	*
***trnY(Tyr)***	*	*	*	*
***trnQ(Gln)***	*	ns	-	-

*: gene present, **-**: gene absent. ns: not sequenced, these genes are expected to be located in the not sequenced part of the mitochondrial genome of *Euplotes crassus*

**Table 2:** Open reading frames (*orf*s) from *Euplotes minuta *that share sequence similarity with *orf*s from *Euplotes crassus, Tetrahymena spp*. and *Paramecium aurelia*.

*Euplotes minuta*	*Euplotes crassus*	***Tetrahymena spp***.	*Paramecium aurelia*
*orf 96*	*orf101*	-	-
*orf-rps3*	*orf-rps3*	*ymf64*	*orf234*
*orf187*	*orf134*	-	-
*orf268*	*orf311*	-	-
*orf78*	*orf129*	-	-
*ccmF*(partial)	*orf197*	-	-
*orf163*	*orf449*(partial)	-	-
*orf259*	*orf449*(partial)	-	-
*orf145*	*orf449*(partial)	-	-
*orf111*	*orf175*	-	-
*nad2*(partial)	*orf147*	-	-
-	*orf45*	-	-
*orf267*	*orf297*	*orf161/ymf74*	*orf178-2/ymf84*
*orf102*	-	-	-
*orf155*	*orf156*	-	-
*rps4*(partial)	*orf141*	-	-
*orf49*	ns	-	-
*orf125*	ns	-	-
*orf380*	ns	-	-
*orf190*	ns	-	-
*orf170*	ns	-	-

Partial: only a part of this gene/orf has significant sequence similarity. ns: these orfs are located in the part of the *Euplotes crassus *mitochondrial genome that has not been sequenced. -: no gene/orf found in this organism with significant sequence similarity. The remaining orfs in *Tetrahymena spp*. or *Paramecium aurelia *do not show significant sequence similarity (not shown).

There are no differences in gene order between the closely related *E. crassus *and *E. minuta*, but their gene order is completely different than that of the *Tetrahymena *species and *P. aurelia *(Figure 2). Only four genes could be found that have a conserved order in all these ciliate species: *rpl2-orf-nad10-rps12*.

### Genes encoding components of the electron transport chain

As shown in Table 1 all mitochondrion-encoded Complex I genes that were found in *T. pyriformis *and *P. aurelia *[4], were also found in *E. minuta *with the exception of *nad6/ymf62 *that was identified as *nad6 *in *T. pyriformis *[3]. The mitochondrial genomes of all sequenced *Tetrahymena *species possess *nad6/ymf62*, which exhibits a significant sequence similarity with *orf*265 *in P. aurelia*.

In all *Tetrahymena *species and in *P. aurelia *the Complex I gene *nad1 *is split into a larger A and a smaller B part, which is located on the opposite strand. In *E. minuta *this gene is also split but located on the same strand. In *E. crassus *the corresponding part of the mitochondrial genome has not been sequenced (Figure 2).

The length of the *nad2 *gene of *T. pyriformis, P. aurelia *and *N. ovalis (var. Bla. Ams) *is almost the same but about 180 amino acids smaller than the *nad2 *gene of *Bos taurus *(cow). In contrast, the *nad2 *genes of both *Euplotes *species have large N terminal extensions. The *nad2 *gene of *E. crassus *has an extension of about 250 amino acids, and the homologous gene of *E. minuta *is around 500 amino acids longer. These extensions have no detectable sequence similarity to each other or to other known genes.

The Complex I gene *nad4L *has been identified by Brunk et al. in *T. thermophila *[3] (named *ymf 58 *in the other mitochondrial genomes of *Tetrahymena *species), in the hydrogenosomal genome of the anaerobic ciliate *N. ovalis *and in both *Euplotes *species (Table 1). It has not been annotated in *Paramecium*, but alignments of *orf113 *in *P. aurelia *with the *Tetrahymena *species and with the *nad4L *gene of *N. ovalis *shows that this *orf113 *is homologous to *nad4L *[10]. The *nad7 *genes of both *Euplotes *species are highly conserved; both have a small N-terminal extension (19 and 36 amino acids, respectively). These extensions are not similar to each other and are not found in other ciliates.

**Figure 5:**
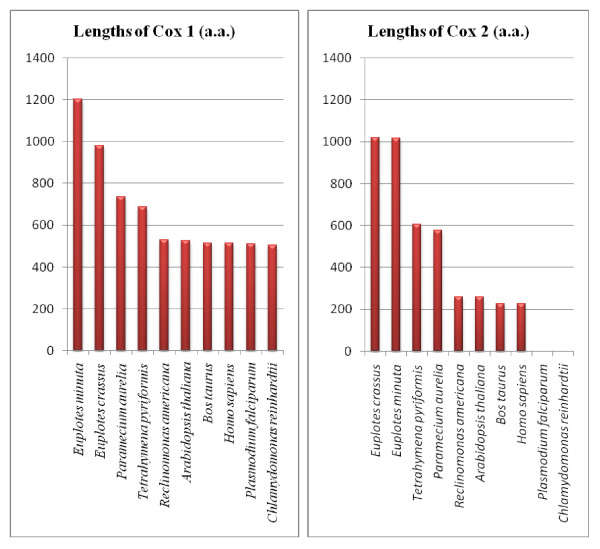
**Length of *cox1 *and *cox2 *open reading frames (in amino acids) from *E. crassus *and *E. minuta *compared to other organisms**.

**Figure 6:**
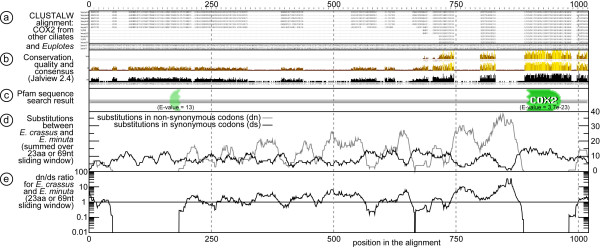
**Alignment analysis of *Euplotes cox2***. a) ClustalW alignment of *cox2 *from other ciliates and *Euplotes*; b) conservation, quality and consensus scores of the multiple alignment in (a) according to Jalview; c) Pfam search result including an insignificant hit to the Cox2 Pfam domain in the N-terminal conserved region of the gene; d) number of non-synonymous (ds) and synonymous (ds) base substitutions observed between *E. minuta *and *E. crassus *per 69 nt sliding window; e) dn/ds ratio based on (d).

The only Complex III gene that is found in the mitochondrial genomes of the *Tetrahymena *species and *P. aurelia *is *cytochrome-b *(*cob*), which has also been identified in both *Euplotes *species (Table 1; Figure 2). The *cob *genes of both *Euplotes *species possess small N-terminal extensions that are not conserved while the remaining part of the gene is very well conserved. The Complex IV genes *cytochrome oxidase 1 *and *2 (cox1 *and *cox2*), are found in all *Tetrahymena *species, in *P. aurelia *and in both *Euplotes *species (Table 1; Figure 2). As shown earlier in *T. pyriformis *and *P. aurelia *both genes contain large (in frame) upstream open reading frames [4]. In *Euplotes *the *cox2 *frames reach extreme lengths, 1021 amino acids in *E. crassus *and 1017 amino acids in *E. minuta *(Figure 5a, b). The insert does not show significant similarity to any known gene, precluding the inference of function and functional constraints by sequence similarity. The sequencing of two *Euplotes *species, however, allows us to assess whether there is any selection on the protein coding sequence by calculating the ratio of non-synonymous over synonymous substitutions (dn/ds) and test for protein sequence conservation. Figure 6a shows the ClustalW alignment [19] between the Cox2 proteins in *Euplotes *and several other ciliate species. There are only two regions that could be unequivocally aligned among all the ciliates and of which the alignment did not depend on the program used [20,21]. These regions are indicated by the high conservation and quality bars in Figure 6. They are also highly conserved between the two Euplotes sequences (dn = 0), and overlap with the regions for which we detected likely sequence similarity with the PFAM Cox2 domain, albeit with an insignificant E-value for the N-terminal part [22]. In contrast, there appear to be less constraints on the primary sequence of the ~700 amino acid in-frame *cox2 *insert (65% identity between the two Euplotes sequences). For *cox1*, we found a similar situation (see Additional file 1: Fig. S1 and Additional file 2:Fig. S2) When the Cox1 protein sequences of both *Euplotes *species are compared with the Cox1 protein sequence of *Bos taurus *a large insert in frame of 380 amino acids was identified between positions 119-120. *T. pyriformis *and *P. aurelia *possess an insert of 271 amino acids in exactly the same position. Furthermore, it seems that the *cox1 *genes of *T. pyriformis *and both *Euplotes *species contain N-terminal extensions of about 40 amino acids. The N-terminal extension in *P. aurelia *is a bit longer, about 83 amino acids. The N-terminal extensions of the *cox1 *gene in both *Euplotes *species and in *P. aurelia *harbour a potential mitochondrial import signal that has been identified by the program Mitoprot [23]. In a recent publication [24] it was observed that latent mitochondrial targeting signals are present on the mitochondrial genomes of *Arabidopsis thaliana *and *Oryza sativa*. It is possible that some of the N-terminal extensions we find in *Euplotes spp*. play a role as latent mitochondrial targeting signals. Alternatively, they could function as an internal localization signal, resulting from a bias in nucleotide alteration, or even hint at the possibility of back-transfer of genes from the nucleus to the organelle [25]. Furthermore, the *cox1 *gene of *E. minuta *possesses a C-terminal extension (267 amino acids) that has not been found in the other ciliates, including *E. crassus*.

Another cytochrome c related gene, *ccmF/yejR*, is also found in both *Euplotes *species (Table 1). It is a cytochrome c assembly protein that encodes the potential catalytic subunit of cytochrome c lyase. There is a large difference in the lengths of the *ccmF/yejR *genes between these ciliates. *T. pyriformis *(513 amino acids) and *E. minuta *(461 amino acids) have a large C-terminal extension. The corresponding extensions in *P. aurelia *(256 amino acids) and *E. crassus *(243 amino acids) are significantly smaller.

Only one Complex V gene, *ATPase 9*, has been identified in *E. minuta*. It is also located on the mitochondrial genomes of *T. pyriformis *and *P. aurelia *(Table 1). The corresponding region in *E. crassus *was not sequenced.

### Ribosomal proteins

Mitochondrial genes encoding mitochondrial ribosomal proteins are common in plants and protists but have never been found in the mitochondrial genomes of animals. Ciliates possess a limited number of ribosomal proteins on their mitochondrial genomes. So far 7 ribosomal proteins have been identified in *E. minuta *(Table 1). Another ribosomal protein, *rpl14 *from *E. crassus*, which is present in all other ciliate mitochondrial genomes, has an N-terminal extension (33 amino acid) that has no significant similarity with other known proteins. Similar extensions were observed for the *rpl16, rps4 *and *rps12 *genes in both ciliates *E. crassus *and *E. minuta *(Table 3). When the mitochondrial genes were examined with the mitochondrial import signal prediction program Mitoprot [23] we found high scoring hits for all ribosomal proteins in *E. minuta *and all ribosomal proteins in *E. crassus *except one. All these ribosomal proteins also contained a predicted cleavage site. Analyses of the mitochondrial ribosomal proteins of *T. pyriformis *and *P. aurelia *indicated that also some of these proteins possessed a potential import signal. An analysis based on the signal prediction programs Predotar [26] and TargetP [27] gave less hits but still identified a significant number of potential mitochondrial import signals (Table 3).

**Table 3:** Importsignal and cleavage-site prediction by Mitoprot of mitochondrion encoded genes.

*gene*	*Euplotes minuta*	*Euplotes crassus*	*Tetrahymena pyriformis*	*Paramecium aurelia*	*Blastocystis* *(DMP)*	*Reclinomonas americana*	*Arabidopsis thaliana*	*Oryza sativa*
*nad1a*	0.3731Y		**0.9380Y**	0.8786Y	0.0535N	0.2041N	0.0304N	0.0318Y
*nad1b*	0.0179N		0.0155N	0.0835N				
*nad2*	0.9057Y*#	0.0229Y	0.1574N	**0.9175Y**	0.1689N	0.0435N	0.1369N	0.0497N
*nad3*	0.1585N		0.0175N	0.1252N	0.1418N	0.0504N	0.0154N	0.0042N
*nad4*	0.0642Y	0.7605N	0.6499N	0.4964N	0.5047Y	0.7783N	0.1300N	0.0805N
*nad4L*	0.0155N	0.0103N	0.0657N		0.3538N	0.2082Y	0.3533Y	0.3009N
*nad5*	0.1896N	0.0983N#	0.3453N	0.7374Y	0.5069N	0.3453Y	0.1255Y	0.1675Y
*nad6*						0.1870N	**0.9123Y**	0.7924N#
*nad7*	0.1782N	0.1914N	0.2221Y#	0.3188N	0.6599Y*#	0.6771N	0.6916N#	0.4186N
*nad8*					0.3912N			
*nad9*	0.8492 Y#	**0.9601Y**	0.6518N	0.1990N	0.9159Y*	0.2870N	0.0587N	0.1915N
*nad10*	0.3770Y*	0.2760Y	0.1351Y	0.2149Y		0.2381N		
*nad11*					0.0456N	0.0214N		
*cob*	0.0081N	0.1385N	0.0323N	0.1088N		0.6913N	0.2913N*#	0.2609N*#
*cox1*	**0.9306N**	**0.9803N**	0.1062N	0.9618N*		0.0144Y*	0.2360N*	0.1908N*
*cox2*	0.0503N	0.0203N	0.4944Y	0.6414Y		0.0327N	0.0176N	0.3447N
*cox3*						0.0600Y	0.0605N	0.0222N
*atp1*							0.2910N	0.1920N
*atp6*							0.2621N	0.0085N
*atp9*	0.7172Y		0.3150N			0.1172Y	0.0397N	0.4941Y
*yejR*	0.4823N	0.1926N*#	0.1009N	**0.9263Y**				
*rps1*						0.6830N		0.0249N
*rps2*						0.0626Y		0.5471Y
*rps3*	**0.9987Y#**	1.000Y*#	0.1184N*	0.0581N	0.6604N	0.1605Y	*0.5611N*	0.4670N
*rps4*	**0.9898Y**	0.9364Y*#				0.7786N	0.8602Y*#	0.9984Y*#
*rps7*						0.1327N	0.1334N	0.2046N
*rps8*					0.6514N	0.5082N		
*rps10*					0.8777N	0.1099N		
*rps11*						0.0295N		
*rps12*	0.9998Y*#	0.9999Y*#	0.9351Y*	**0.9683Y**	0.9975Y*	0.3302N	0.2447N	0.1545N
*rps13*			0.1107N	0.6322N	0.9347N*	0.7738N*		0.3213Y
*rps14*			0.9868Y*	0.0604N	0.1811N	0.0376N*		
*rps19*			0.9934N*		0.1278N	0.8812Y*		0.7065N*#
*rpl1*						0.0921N		
*rpl2*	**0.9997Y**	**0.9982Y**	0.9975Y*	**0.9854Y#**	0.4959N*	0.4860Y*	0.9974Y*#	0.9972Y*#
*rpl5*						0.5942N	0.0114N	0.0437N
*rpl6*			0.5948N		0.6851N*	0.3297N		
*rpl10*						0.8245N		
*rpl11*					**0.9694N**	0.0835N		
*rpl14*	0.9298Y*#	0.0484N	0.2054N	0.0817N	0.4278Y	0.1262N		
*rpl16*	0.9982Y*#	0.9655Y*#	0.9947Y*#	0.8770N	0.7748Y*#	0.4554Y	0.9809Y*	0.7261Y*
*rpl18*						0.5305N*		
*rpl19*						0.6495Y#		
*rpl20*						**0.9939Y#**		
*rpl27*						0.6704Y		
*rpl31*						0.6276N		
*rpl32*						0.9946N*#		
*rpl34*						0.9508Y*		

Bold face: significant import probability due to Mitoprot prediction. *italics*: genes used by Ueda et al. [24]. Y: cleavage site predicted with Mitoprot. N: no cleavage site predicted with Mitoprot. Putative N-terminal targeting sequences for ribosomal proteins were also calculated using the programs Predotar [26] and TargetP [27] *: these proteins could be imported into mitochondria according to Predotar. #: these proteins could be imported into mitochondria according to TargetP.

### tRNA genes

Among eukaryotes the number of mitochondrial-encoded tRNA genes varies from 26 tRNAs in *Reclinomonas americana *to zero in apicomplexa [28]. Seven different tRNA genes were identified in the mitochondrial genome of *E. minuta (trnE, trnF, trnH, trnM, trnY, trnQ *and *trnW*) in contrast to only four such tRNA genes in *P. aurelia *(*trnF, trnM, trnW *and *trnY) *(Table 1) [1]. In *E. crassus *only 5 tRNA genes were identified. Also, in *T. pyriformis *a set of seven tRNA genes were identified i.e. *trnE, trnF, trnH, trnL, trnM, trnW *and *trnY *[4]. The mitochondrial-encoded tRNA for Glutamine (*trnQ*) seems to be unique for *Euplotes*, since it was not identified in either *T. pyriformis *or in *P. aurelia; trnL *is duplicated in *T. pyriformis*. Two different programs (tRNAscan-SE and ARAGORN) did not detect a tRNA for tryptophan (W); instead, this tRNA was identified as a tRNA for selenocystein. Recently however, the presence of *trnW *in the mitochondrial genome of *E. crassus *was experimentally confirmed by Turanov et al. [29].

### Open reading frames

Additional 17 *orf*s have been identified in *E. minuta *and 13 *orf*s in *E. crassus *(Table 2). One *orf *(*rps3*) of *E. minuta *and *E. crassus *has, after BlastX and BlastN searches, detectable sequence similarity with *orf*s from *T. pyriformis *and *P. aurelia *(*ymf64/orf234*). In *T. thermophila *the gene *ymf64 *has been identified as a putative ribosomal protein, based on physicochemical parameters of the predicted protein [3]. Comparison of an alignment of the *ymf64 *homologs in the ciliates with the Hidden Markov Models (HMMs) in PFAM, using the sensitive profile-profile based homology detection tool HHsearch [30] indicates that *ymf64 *exhibits significant sequence similarity with the C-terminal domain of the ribosomal protein S3 (P < 2.1 E-5, Additional file 3: Fig. S3). An HMM of the genes that are currently annotated as *rps3 *in the *Tetrahymena *species and in *P. aurelia *indicated that they are homologous to the N-terminal domain of the ribosomal protein S3 (P < 2.1 E-5). The gene length of *ymf64 *in *T. thermophila *is 330 amino acids; in *P. aurelia *(*orf234*) it has a length of 234 amino acids. The orthologous *Euplotes *genes are much larger (767 and 768 amino acids, respectively). We could not detect significant sequence similarity of the S3 N-terminal domain to any of the *Euplotes *sequences.

For the remaining 16 *orfs *in *E. minuta *and 12 in *E. crassus *no homologous genes were found using BlastX and BlastN searches. However, one of these, *orf267(orf 297 *in *E. crassus)*, which is part of the conserved region of four genes in *Euplotes spp*., is weakly conserved when compared to *orf161/ymf74 *in *T. pyriformis *and *orf178-2/ymf84 *in *P. aurelia *(Table 2)

### Mitochondrial ribosomal RNA genes

The mitochondrial large and small subunit ribosomal RNA genes in five *Tetrahymena *species and in *P. aurelia *are split into two pieces [4]. In all these *Tetrahymena *species the *rnl *gene is duplicated. Analysis of the mitochondrial genomes of *E. minuta *and *E. crassus *by BlastN identified the regions were the *rnl *and *rns *genes are situated. The *rnl *gene in *Euplotes *species is not duplicated as in the *Tetrahymena *species. Even by sensitive Smith-Waterman queries [31] with selected parts of the *rnl *and *rns *sequences from other ciliate species, we did not find any indication that these genes were split in *Euplotes *(not shown). Both, the region containing the putative *rnl *and the region containing the putative *rns*, have significant sequence similarity to the *rnl *and the *rns *of the published mitochondrial ciliate ribosomal RNAs. Nevertheless, the regions of significant sequence similarity do not cover the complete published ribosomal RNAs, prohibiting complete sequence alignment and therewith assessment as to whether these RNAs are complete or interrupted. As expected, a 5S rRNA gene could not be identified.

### Genetic code

Analysis of the codon usage as described in the Methods section confirmed that both *Euplotes *species use the protozoan mitochondrial code, with TGA encoding tryptophan. There are a few spurious predictions (TCG, ATG and ACC in *E. crassus *and TAG, ATT, ATG and ACC in *E. minuta*), but for all these cases we find the correct translation at an almost equal score. The prediction that TAG would code for a serine in *E. minuta *is only based on a single aligned occurrence of the codon, caused possibly by a sequence error or a misalignment (not shown).

## Conclusion

When the mitochondrial genome of *T. pyriformis *was published and compared with that of *P. aurelia*, it seemed that the mitochondrial gene order in ciliates was very well conserved [4]. With the determination of the mitochondrial genome of a third ciliate genus, belonging to a completely different taxon, we have shown that the gene order in mitochondrial genomes of ciliates can be very different while a similar set of genes is conserved. Also the linearity of the mitochondrial chromosomes is conserved. This might suggest that monomeric linear mitochondrial chromosomes, which are relatively rare among protozoa and animals [32], are characteristic for ciliates. This possibility is corroborated by the observation that also species belonging to the sister taxon apicomplexa possess linear mitochondrial chromosomes [33]. However, it should be noted that among yeasts even the mitochondrial genomes of closely related species differ with respect to their linearity/circularity [32].

From the 17 unidentified open reading frames in the mitochondrial genome of *Euplotes minuta *two could be found with significant sequence similarity to *T. pyriformis *and *P. aurelia *(Table 2). This contrasts with the situation in *T. pyriformis *and *P. aurelia *where 13 out of the 22 unidentified open reading frames in *T. pyriformis *were also found in *P. aurelia *[4]. One of these orfs, *ymf64*, has now, with the aid of the *Euplotes *sequences and profile based homology searches been shown to be significantly similar to a known protein domain, the C-terminal part of the Rps3 protein. This suggests that with the sequencing of more mitochondrial genomes of the ciliates also for other orfs sequence similarity might be detected with known mitochondrial genes.

One of the rare mitochondrial features present in the mitochondrial genomes of *T. pyriformis *and *P. aurelia *is a split *nad1 *gene. This split gene has also been identified in *Euplotes *and thus seems to be specific for a large group of (maybe all) ciliates.

One of the most striking differences between the mitochondrial genomes of *Euplotes *and those of *Tetrahymena *species and *P. aurelia *is the presence of a large repeat region in the middle of the mitochondrial genome of both *Euplotes *species that seems to be used as a bi-directional transcription start. No such repeat was found in *Tetrahymena *species and *P. aurelia *and, in contrast to *Euplotes*, the transcription direction changes several times.

Another striking feature of the genes in the mitochondrial genome of *Euplotes *species is the presence of very large open reading frames. Most of these large *orf*s contain N-terminal extensions, but in some cases, like the *cox1 *and *cox2 *genes, large inserts in frame cause this effect. Such inserts in frame were also detected in *Tetrahymena sp*. and *P. aurelia*. Surprisingly, all of the N-terminal extensions of genes encoding ribosomal proteins of *Euplotes minuta *contain a potential targeting signal for import into mitochondria. This is the first report identifying such import signals in mitochondrial-encoded genes in organisms other than plants.

Sequencing and analyzing the mitochondrial genomes of *E. crassus *and *E. minuta *shows that the mitochondrial genomes of ciliates are rearranged more extensively than previously thought. Sequencing of the mitochondrial genome of *E. minuta *also did not provide any evidence for the presence of a slightly deviating, alternative genome that might be expected for the two morphs of mitochondria observed in this species. Studying these mitochondrial genomes has provided additional information about the evolution of mitochondria in general and in particular about the evolution of the elusive hydrogenosomal genome of *Nyctotherus ovalis *[10], which appeared to be more related to the mitochondrial genome of *Euplotes *than to those of *Paramecium *and *Tetrahymena*.

## Methods

*E. minuta *cells were collected in 2005 in the Mediterranean sea near Stareso, Corsica, France (Em. S1, *E. minuta *Stareso1), cultured in the laboratory in artificial sea water obtained from the Botanical and Zoological Garden Stuttgart (Wilhelma) and fed with *Klebsiella minuta *grown on nutrient agar. For the isolation of DNA, a concentrated sample of living cells was mixed with 8 M guanidiniumchloride. A 10:1 mixture with 1 M phosphate buffer pH 7.0 was made, adsorbed on a hydroxyapatite (Biorad, bio-gel HTP) column (1 cm × 0.4 cm) and washed with 4 M guanidiniumchloride, 100 mM phosphate buffer pH = 7.0, followed by washing with 4 M guanidiniumchloride, 200 mM phosphate buffer pH = 7.0. Subsequently, the bulk of DNA was eluted with 4 M guanidiniumchloride 500 mM phosphate buffer pH = 7.0. The DNA was diluted with 1 volume water and precipitated with 10 v/v% 3 M sodiumactetate pH = 5.2 and 50 v/v % propanol-2 for 10 minutes at room temperature. After precipitation and washing the pellet was air dried. Finally, the DNA pellet was dissolved in DEPC treated water (Invitrogen).

The dissolved DNA was loaded on a pulsed field agarose gel (1% agarose type II medium EEO, Sigma) and run at 170 V (145 mA) ramping from 2.5 s - 25 s for 16 hours PFGE with a LKB 2015 Pulsaphor plus control unit.

The band just below the first band of the lambda marker (Figure 3) was cut out and the DNA extracted. The position of the mitochondrial band on pulsed field gel is a clear indication of a linear mitochondrial genome. Circular mitochondrial genomes of this size should run much faster in the gel. The DNA of the band was digested with Sau 3A and then size fractionated on an agarose gel. The DNA from these fractions was isolated from the gel, ligated in pUC-18 digested with BamH1 and transformed in E. coli DH101B cells. The titre of the library was 1.12 × 10^5^. From this library, plasmid DNA from 288 different colonies was sequenced with an ABI prism 3730 online capillary sequencing machine and the mitochondrial genome was assembled as described below. The gene library was constructed by Genterprise, Mainz, Germany.

*E. crassus *was collected from shallow coastal waters of the sandy beach of Porto Recanati (43° 26' N, 13° 40' E) on the Italian Adriatic Coast, 50 km south of Ancona, July 1984 and cultured in the laboratory in artificial sea water (NaCl 465 mM, KCl 10 mM, MgCl_2_.6H_2_O 24.8 mM, MgSO_4_.7H_2_O 28.1 mM, CaCl_2 _10.4 mM, NaHCO_3 _2.4 mM pH 8.0).

Initially, a culture was kept in artificial seawater in an Erlenmeyer flask and fed with a small piece of raw beef. Alternatively, a set of 200 ml tissue flasks was first siliconized, filled with approximately 50 ml of artificial seawater, and inoculated with *E. crassus *cells. These cultures were fed with HB101 *E. coli *cells.

Total DNA of *E. crassus *was isolated by dissolving cells in 8 M guanidiniumchloride and purification by hydroxyapatite as described above for *E. minuta*.Four fragments of different mitochondrial genes were obtained by PCR with degenerated primers on this DNA, i.e. primers directed against the ribosomal genes *rnl *(5'-GTCAAGAGAGAAACAGC-3', 5'-GCATAGGGTCTTCCCGTC-3'), *rns *(5'-TGTGCCAGCAGCCGCGGTAA-3', 5'-TCCCMTACCRGTACCTTGTGT-3') and the complex I genes *nad7 *(5'-TTCGGWCCHCARCAYCCHGC-3', 5'-CTRTCRACYTCWCCRAARAC-3') and *nad10 *(5'-TTYGGHYTNGCHTGHTG-3', 5'-ARDGCYTCDSWDGTDGGDGGDCA-3') On these gene fragments primers for long range PCR were developed and long range PCR with LA-Taq-polymerase (5 U/μl) (Takara bio inc.) was performed. The long range PCR products were digested with different restriction enzymes, subcloned in pUC-18 (Sigma) or in pGEM-T easy (Promega) and sequenced. Sequencing was performed at the DNA diagnostics centre of the Nijmegen University Medical Center using M13 forward and reverse primers.

All sequences have been submitted to NCBI GenBank. The GenBank accession-numbers are for *E. minuta *GQ903130 and for *E. crassus *GQ903131. The protein identifiers are displayed in additional file 4.

### Analysis of the sequence data

Sequences were edited using chromas Lite 2.01 http://www.technelysium.com.au The edited sequences were assembled using BioEdit version 7.0.9.0 [34]. Open reading frames were identified with *orf *Finder http://www.ncbi.nlm.nih.gov/gorf/gorf.html. tRNAs were identified with tRNAscan-SE http://lowelab.ucsc.edu/tRNAscan-SE/ and ARAGORN [35]. Sequence similarity searches of deduced amino acid sequences were performed with BLASTX and BLAST2 [36]. The nucleotide sequence similarity searches were conducted with BLASTN (NCBI) and FASTA (EMBL-EBI). Import signal prediction was done with Mitoprot [23], Predotar [26] and TargetP [27]. Alignments were made with ClustalX2 and ClustalW [37]. The program Nucleic Acid Dot Plots http://www.vivo.colostate.edu/molkit/dnadot/index.html was used for identifying repeat structures.

The sequences for the 18s rRNA phylogeny were aligned using the SINA Webaligner http://www.arb-silva.de/aligner, which aligns them in accordance with the ARB/SILVA rRNA alignment [38] which is based on a secondary structure model [39]. Subsequently we used Gblocks [40] to identify reliably aligned parts, using the default settings except that we did not require the coverage for every position to be 100%, but rather 80%. We then used PhyML v3.0.1 (HKY85 model, optimised equilibrium frequencies, estimated ts/tv ratio, estimated proportion of invariable sites, 4 substitution rate categories, estimated gamma distribution parameter, NNI tree topology search, 100 bootstrap iterations [41]) to obtain the phylogeny.

The genetic code used for the translation of the *Euplotes *mitochondrial DNA was derived using the standard genetic code for translation of the complete DNA sequence in 6 frames, and searching the resulting protein sequences for conserved Pfam-fs protein domains [42] using HMMPFAM [30]. The amino acid frequencies provided by the Pfam HMM profiles were then used to predict the translation of each codon. Averaging over all aligned occurrences of the codon, the highest scoring (i.e. most often aligned) amino acid was predicted to be the translation of the codon *in vivo*.

## Authors' contributions

RdG participated and coordinated cloning, sequencing and analyzing of both mitochondrial genomes and drafted the manuscript. BD analyzed the genetic code and performed the bioinformatic analysis. TvA participated in sequencing and analyzing the mitochondrial genomes. HvZ and MBH cloned and sequenced parts of the mitochondrial genome of *Euplotes crassus*. JK participated in cloning, sequencing and analyzing the mitochondrial genome of *Euplotes minuta*. HDG cultivated *Euplotes minuta *and provided cells for PFGE. MH supervised sequence analysis and participated in drafting the manuscript. JH initiated and coordinated the study and participated in drafting the manuscript. All authors read and approved the final version of the manuscript.

## Supplementary Material

Additional file 1 **Figure S1**. Multiple sequence alignment of the N-terminal part of Cox1. Click here for file

Additional file 2 **Figure S2**. Multiple sequence alignment of the C-terminal part of Cox1. Click here for file

Additional file 3 **Figure S3**. Multiple sequence alignment of the C-terminal part of the ribosomal protein S3. Click here for file

Additional file 4 **Protein identifiers**. Accession numbers of mitochondrion-encoded proteins. Click here for file
