# Supplementary material for: The mitochondrial genomes of the ciliates Euplotes minuta and Euplotes crassus
Source: BMC Genomics. 2009 Nov 6;10:514. doi: 10.1186/1471-2164-10-514 (PMC2779199; doi:10.1186/1471-2164-10-514)
Supplement: Additional file 1 — Figure S1. Multiple sequence alignment of the N-terminal part of Cox1. [file 1471-2164-10-514-S1.pdf]

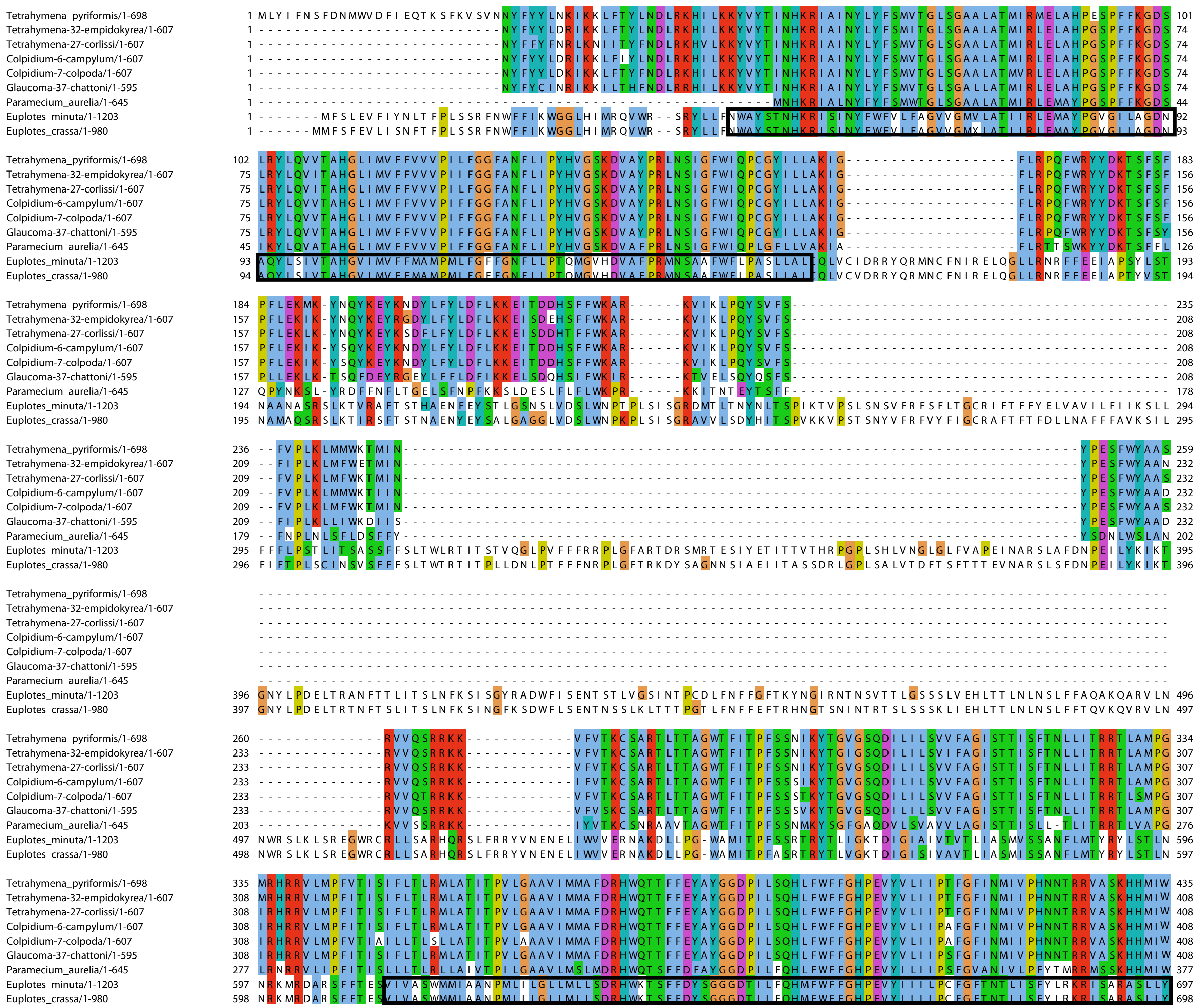

Tetrahymena_pyriformis/1-698
Tetrahymena-32-empidokyrea/1-607
Tetrahymena-27-corlissi/1-607
Colpidium-6-campylum/1-607
Colpidium-7-colpoda/1-607
Glaucoma-37-chattoni/1-595
Paramecium_aurelia/1-645
Euplotes_minuta/1-1203
Euplotes_crassa/1-980
