# Supplementary material for: The mitochondrial genomes of the ciliates Euplotes minuta and Euplotes crassus
Source: BMC Genomics. 2009 Nov 6;10:514. doi: 10.1186/1471-2164-10-514 (PMC2779199; doi:10.1186/1471-2164-10-514)
Supplement: Additional file 3 — Figure S3. Multiple sequence alignment of the C-terminal part of the ribosomal protein S3. [file 1471-2164-10-514-S3.pdf]

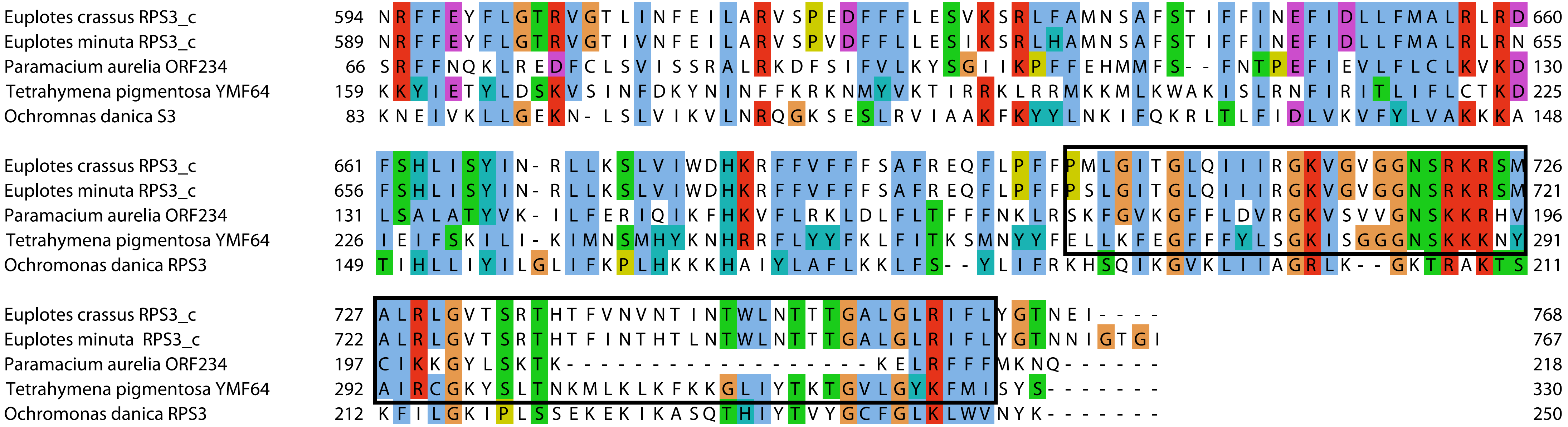
Euplotes crassus RPS3_c 594 NRFFEYFLGTRVGTLINFEILARVSPEDFFFLESVKSRLFAMNSAFSTIFFINEFIDLLFMALRLRD 660
Euplotes minuta RPS3_c 589 NRFFEYFLGTRVGTIVNFEILARVSPVDFFLLESIKSRLHAMNSAFSTIFFINEFIDLLFMALRLRN 655
Paramacium aurelia ORF234 66 SRFFNQKLREDFCLSVISSRALRKDFSIFVLKYSGIIKPFFEHMMFS--FNTPEFIEVLFLCLKVKD 130
Tetrahymena pigmentosa YMF64 159 KKYIETYLDSKVSINFDKYNINFFKRKNMYVKTIRRKLRRMKKMLKWAKISLRNFIRITLIFLCTKD 225
Ochromnas danica S3 83 KNEIVKLLGEKN-LSLVIKVLNRQGKSESLRVIAAKFKYYLNKIFQKRLTLFIDLVKVFYLVAKKKA 148
Euplotes crassus RPS3_c 661 FSHLISYIN-RLLKSLVIWDHKRFFVFFFSAFREQFLPFFPMLGITGLQIIIRGKVGVGGNSRKRSM 726
Euplotes minuta RPS3_c 656 FSHLISYIN-RLLKSLVIWDHKRFFVFFFSAFREQFLPFFPSLGITGLQIIIRGKVGVGGNSRKRSM 721
Paramacium aurelia ORF234 131 LSALATYVK-ILFERIQIKFHKVFLRKLDLFLTFFFNKLRSKFGVKGFFLDVRGKVSVVGNSKKRHV 196
Tetrahymena pigmentosa YMF64 226 IEIFSKILI-KIMNSMHYKNHRRFLYYFKLFITKSMNYYFELLKFEGFFFYLSGKISGGGNSKKKNY 291
Ochromonas danica RPS3 149 TIHLLIYILGLIFKPLHKKKHAIYLAFLKKLFS--YLIFRKHSQIKGVKLIIAGRLK--GKTRAKTS 211
Euplotes crassus RPS3_c 727 ALRLGVTSRTHTFVNVNTINTWLNTTTGALGLRIFLYGTNEI---- 768
Euplotes minuta RPS3_c 722 ALRLGVTSRTHTFINTHTLNTWLNTTTGALGLRIFLYGTNNIGTGI 767
Paramacium aurelia ORF234 197 CIKKGYLSKTK------------------KELRFFFMKNQ------ 218
Tetrahymena pigmentosa YMF64 292 AIRCGKYSLTNKMLKLKFKKGLIYTKTGVLGYKFMISYS------- 330
Ochromonas danica RPS3 212 KFILGKIPLSSEKEKIKASQTHIYTVYGCFGLKLWVNYK------- 250
