# Supplementary material for: The mitochondrial genomes of the ciliates Euplotes minuta and Euplotes crassus
Source: BMC Genomics. 2009 Nov 6;10:514. doi: 10.1186/1471-2164-10-514 (PMC2779199; doi:10.1186/1471-2164-10-514)
Supplement: Additional file 4 — Protein identifiers. Accession numbers of mitochondrion-encoded proteins. [file 1471-2164-10-514-S4.doc]

| ***Euplotes minuta*** |  |  | ***Euplotes crassus*** |  |
| --- | --- | --- | --- | --- |
| gene | protein_identifier |  | Gene | protein identifier |
| NAD5 | ACX30940 |  | NAD5 | ACX30976 |
| ORF145 | ACX30941 |  | ORF449 | ACX30977 |
| ORF259 | ACX30942 |  | ORF197 | ACX30978 |
| ORF163 | ACX30943 |  | ccmF/jeyR | ACX30979 |
| ccmF/jeyR | ACX30944 |  | cytb | ACX30980 |
| cytb | ACX30945 |  | ORF129 | ACX30981 |
| ORF78 | ACX30946 |  | COX2 | ACX30982 |
| COX2 | ACX30947 |  | RPL14 | ACX30983 |
| RPL14 | ACX30948 |  | COX1 | ACX30984 |
| COX1 | ACX30949 |  | NAD4L | ACX30985 |
| NAD4L | ACX30950 |  | ORF311 | ACX30986 |
| ORF187 | ACX30951 |  | ORF134 | ACX30987 |
| RPS3 | ACX30952 |  | RPS3 | ACX30988 |
| ORF96 | ACX30953 |  | ORF101 | ACX30989 |
| NAD9 | ACX30954 |  | NAD9 | ACX30990 |
| ORF111 | ACX30955 |  | ORF175 | ACX30991 |
| NAD2 | ACX30956 |  | ORF147 | ACX30992 |
| RPL16 | ACX30957 |  | NAD2 | ACX30993 |
| NAD4 | ACX30958 |  | ORF45 | ACX30994 |
| RPS12 | ACX30959 |  | RPL16 | ACX30995 |
| NAD10 | ACX30960 |  | NAD4 | ACX30996 |
| ORF267 | ACX30961 |  | RPS12 | ACX30997 |
| RPL2 | ACX30962 |  | NAD10 | ACX30998 |
| ORF102 | ACX30963 |  | ORF267 | ACX30999 |
| ORF155 | ACX30964 |  | RPL2 | ACX31000 |
| RPS4 | ACX30965 |  | ORF156 | ACX31001 |
| NAD7 | ACX30966 |  | ORF141 | ACX31002 |
| ORF49 | ACX30967 |  | RPS4 | ACX31003 |
| NAD1b | ACX30968 |  | NAD7 | ACX31004 |
| ORF125 | ACX30969 |  |  |  |
| ORF380 | ACX30970 |  |  |  |
| ATP9 | ACX30971 |  |  |  |
| ORF190 | ACX30972 |  |  |  |
| ORF170 | ACX30973 |  |  |  |
| NAD3 | ACX30974 |  |  |  |
| NAD1a | ACX30975 |  |  |  |
